# Recommended patient information sheet on the impact of haematopoietic cell transplantation on sexual functioning and sexuality

**DOI:** 10.3332/ecancer.2019.987

**Published:** 2019-12-12

**Authors:** Corien M Eeltink, Luca Incrocci, Irma M Verdonck-de Leeuw, Sonja Zweegman

**Affiliations:** 1 Amsterdam University Medical Center, Cancer Center Amsterdam, Department of Hematology, Vrije Universiteit Amsterdam, 1081 HV Amsterdam, The Netherlands; 2 Department of Radiation Oncology, Erasmus MC, 3015 GD Rotterdam, The Netherlands; 3 Cancer Center Amsterdam, Department of Clinical Psychology, Vrije Universiteit Amsterdam, 1081 HV Amsterdam, The Netherlands; 4 Department of Otolaryngology, Amsterdam University Medical Centers (Amsterdam UMC), location VUmc, 1105 AZ Amsterdam, The Netherlands; 5 Cancer Center Amsterdam, EMGO+ Institute, 1081 HV Amsterdam, The Netherlands

**Keywords:** haematopoietic cell transplantation, sexual function, sexuality, patient information, communication

## Abstract

Sexual concerns are common after haematopoietic cell transplantation (HCT). Exposure to total body irradiation (TBI), alkylating agent and graft versus host disease (GvHD) can all affect sexual function, leading to problems in sexual desire, arousal and the orgasm phase of the sexual response cycle. In high-risk haematological malignancies, such as acute leukaemia and myelodysplastic syndromes, HCT often offers the highest chance for long-term survival. In addition, these haematological diseases and HCT can have an impact on body image, self-esteem, (sexual) relationship and psychosocial factors, all of which are able to affect sexuality and sexual function. Five years post HCT, 80% of the female survivors and 46% of the male survivors report sexual dysfunction.

It has been shown that these patients cope better after having discussed sexual health. While healthcare providers (HCPs) have the responsibility to address sexual issues, it has been demonstrated that 48%–82% HCT recipients reported not having discussed sexual issues with their HCPs and that only one-third of the HCPs routinely discussed sexual issues with their patients. HCPs describe a lack of knowledge and being uncomfortable with the topic as the most important reasons for not addressing sexual functioning. Even so, it would help >90% HCPs if the patient initiated discussing sexual issues. However, to empower patients addressing sexual issues, adequate comprehensive patient information is needed. In an effort to better meet the patients’ need, a patient information sheet: ‘Information for patients undergoing Hematopoietic Cell Transplantation: the impact of the disease and treatment on sexual function and sexuality’, has been created.

In this review, we describe what is known about the impact of HCT on sexual function and briefly the management of sexual problems.

## Introduction

Recent improvements in the treatment of haematological malignancies have increased survival rates and life expectancy. This raises the important question whether and to what extent future (sexual) life is hampered by the side effects of cancer treatment, as sexual dysfunction is of importance for the quality of life of patients [1–3]. Sexual dysfunction is common in the general population, with 40%–45% of adult women, and 20%–30% of adult men, reporting at least one sexual dysfunction [4]. Apart from cancer-treatment-induced sexual dysfunction, risk factors for developing sexual dysfunction are older age, inferior general health status, the presence of comorbidities (e.g. diabetes mellitus, cardiovascular disease, urinary tract infections, psychiatric or psychological disorders) and socio-demographic factors such as being female and having no committed partner [5, 6]. The pathophysiology of sexual dysfunction is heterogeneous, ranging from biological (e.g. vascular, hormonal, neurological, urological, iatrogenic, psychiatric, obesity or poor health) and psychological (e.g. emotional problems, depression and anxiety) to social causes (e.g. sexual abuse, alcohol/tobacco/opioids/ recreational drug abuse, marital problems, difficulty talking about the sexual relationship with the partner, no sexual partner, sexual dysfunction in the partner, low education and unemployment) [4, 7, 8]. Cancer treatment, especially haematopoietic cell transplantation (HCT), is known to affect all these domains, which will be discussed hereafter [9–15].

Patients and their partners want information regarding the effects of illness, treatment and disability on sexuality and intimacy [15, 16]. This (unfulfilled) information need and the attitude of the healthcare providers (HCPs) towards addressing sexuality [13, 17, 18] have heightened the need for easy available information for patients and their partners. In an effort to better meet this need, a patient information sheet: ‘Information for patients undergoing Hematopoietic Cell Transplantation: the impact of the disease and treatment on sexual function and sexuality’, has been created. Topics cover aspects that may affect sexuality and intimacy. Our aim is to make the patient information on sexuality accessible and understandable to patients and their partners. It is hypothesised that this information may facilitate the patient in the discussion of expected sexual problems with their HCP, where necessary.

## The impact of haematopoietic cell transplantation on sexual function

Sexual problems following autologous and allogeneic HCT are common. In high-risk haematological malignancies, such as acute leukaemia and myelodysplastic syndromes, HCT often offers the highest chance for long-term survival. In order to identify literature with respect to sexual function and HCT, a comprehensive search was carried out from the bibliographic databases PubMed and EMBASE from inception till February 20, 2018. Search terms included controlled terms from MeSH in PubMed and Ebsco for CINAHL/PsycINFO. The full search can be retrieved from the corresponding author. To understand sexual dysfunction during and after HCT, we included only longitudinal studies, and three prospective studies were identified [12–14]. Humphreys *et al* [13] showed decreased sexual activity among HCT survivors at 1 and 3 years post HCT compared to pre-HCT. About 60% were sexually active before HCT and 40% at 3 years post HCT. Problems with sexual desire ranged between 33% and 78% and arousal difficulties between 22% and 78%. Women are more likely than men to report sexual difficulties [13]. In addition, Humphreys *et al* [13] examined sexual functioning and communication regarding this issue with their healthcare provider in recipients of allogeneic stem cell transplantation from pre-transplant to 1 and 3 years post-transplant. In addition, those patients who discussed their condition with an HCP reported better sexual function.

In the prospective case-matched controlled study by Syrjala *et al* [12], the sexual function of 161 survivors was evaluated from pre-transplant up to 5 years post HCT. In addition, sexual function in this population was compared with controls from the general population. Five years after treatment, 80% of female survivors versus 61% of female controls and 46% of male survivors versus 21% of male controls reported sexual dysfunction. While the sexual function of female survivors was not improved at 5 years post HCT, the sexual function of the male survivors was improved at 2 years, however, not to the level of sexual function reported by controls. The aetiology is not exactly known; however, the fact that hypogonadism can recover in men, which is not very likely in women, may play an important role [19]. In addition, in general, women appear to report sexual difficulties more often [4].

Wong *et al* [14] found that chronic graft versus host disease (GvHD) in both genders contributed negatively to sexual dysfunction and dissatisfaction during the 3 years following HCT. Both men and women with chronic GvHD reported a negative impact on several domains of sexual function, and in addition, women with chronic GvHD reported significantly poorer sexual satisfaction.

In addition, other reports indicated that both disease and treatment can have an impact on body image, self-esteem, (sexual) relationships and psychosocial factors, all of which can negatively impact intimacy, sexuality and sexual function [7, 20].

## The causes of sexual dysfunction in haematopoietic cell transplantation survivors

HCT is associated with acute and chronic side effects that can result in alterations in sexual functioning. Acute side effects of chemotherapy and total body irradiation (TBI; e.g. nausea, vomiting, hair loss and fatigue) can induce loss of sexual desire [21]. Several anti-cancer drugs are known to affect the biology of the gonadal function and in HCT survivors alkylating agents are suggested to be the main cause of sexual dysfunction. In 99% of female and 92% of male HCT survivors, premature menopause [22–24] and hypogonadism [23, 25, 26] are observed [27]. Problems with sexual interest/desire may be explained by the impact on testosterone—a driver of sexual desire. Premature menopause can lead to vaginal and/or vulvar atrophy, causing continuous discomfort and/or pain, and vaginal dryness during sexual activity, negatively affecting interest and desire. TBI is similarly toxic for gonadal function, but can also impair peripheral nerves and pelvic blood flow [28]. Physical sexual arousal (e.g. erectile function and vaginal lubrication) is mainly guided by circulation and good neural synapses. When one of these is disrupted, men can experience difficulties in developing or maintaining an erection during sexual activity while women can experience difficulty in becoming lubricated.

In the long term, GvHD [14, 29, 30] may also lead to sexual dysfunction with genital GvHD directly impacting sexual function. The first presentation of male genital GvHD is often dyspareunia (difficult or painful sexual intercourse) or urinary difficulties. Inflammatory and non-inflammatory genital skin changes with erectile dysfunction are significantly more frequent in patients with genital GvHD: 80% with genital GvHD versus 36% in patients with non-genital GVHD [30]. For women, the first complaint of genital GvHD is also dyspareunia or urinary difficulties often with similar symptoms (vulvovaginal dryness, pruritis, burning, pain, dysuria, dyspareunia and at times, bleeding) and genital atrophy due to oestrogen deficiency [20].

## Communication about sexual issues

It is clear that HCT survivors cope better after having discussed sexual health [13]. In addition, early recognition and management of sexual dysfunction can lead to improved sexual function and quality of life for HCT survivors and their partners. HCPs have the responsibility to address sexual issues. Nevertheless, sexual dysfunction is an issue that HCPs, as well as patients, find difficult to discuss. A survey among HCPs of the European Society for Blood and Marrow Transplantation (EBMT) showed that a lack of knowledge and being uncomfortable with the topic contributed to avoiding discussions on sexual dysfunction. The majority of HCPs (>90%) were reluctant to discuss the topic feeling it would be more appropriate if the patients themselves initiated the discussion regarding sexual issues [18]. Most HCPs felt they had not received appropriate training to address sensitive issues like sexual health [31], were often too embarrassed to ask about sexual concerns or thought that the decline of sexual function is a normal event. In general, sexual issues are not routinely discussed with HCT recipients with 48%–82% of the recipients reporting not having discussed sexual issues with their HCP [13, 17, 32]. Therefore, it is not surprising that many patients and their partners were disappointed by the lack of information, support and practical strategies provided by healthcare professionals [15, 16].

It is not only important that survivors are informed about the impact that HCT might have on both sexuality and sexual function, information should also be tailored to the individual. For some, knowing that sex and reproduction are affected is sufficient, and they do not always need support in case of sexual dysfunction. However, other survivors need to know whether treatment or support is available. Apart from extensive patient information, HCT recipients should be questioned about urinary symptoms and sexual health. Part of the regular patient follow-up can identify patients with genital GvHD resulting in timely referral for adequate management [30].

## Treatment strategies for sexual dysfunction

Treatment of sexual dysfunction is dependent on the cause(s). Although the main focus of this review article is supplying patient information, we provide a concise overview of management suggestions and interventions.

Within HCT, the efficacy of the majority of the biological interventions for sexual problems has not yet been demonstrated [14]. However, several treatment options for sexual dysfunction are said to be worth trying [21]. As most patients perceive some changes in sexuality and sexual function after HCT, they need to rebuild their sexual life. Consequently, when patients report sexual difficulties, it is important to ask about the partners’ feelings and whether the couple are able to discuss this within the relationship. If necessary, communication about the sexual relationship with the partner should be promoted. In case of fatigue, pain or other complaints, the patient should be advised to rest or to use analgesia before sexual activity, or other appropriate advice regarding symptom management should be given.

In the case of sexual problems due to hypogonadism and erectile dysfunction, testosterone and phosphodiesterase type 5 inhibitor (PDE5 inhibitors) can improve sexual function [28, 33–35]. For treatment or prevention of postmenopausal vaginal atrophy, various options are available, such as hormonal replacement therapy (systemic and topical) [36]. Vaginal lubricants and moisturisers are available to prevent or minimise dryness and pain during sexual activity in case of vaginal atrophy or decreased vaginal lubrication [36].

There are several healthcare professionals and disciplines that may facilitate patient management.

- Nurse practitioner, clinical nurse specialist.
- Social worker or psychologist in case of relational difficulties.
- Pelvic physiotherapy in case of dyspareunia or vaginal pain when having sexual intercourse and when vaginal lubricants do not give relief.
- Male/female sexologist in case of need for intensive treatment after all specific suggestions have been tried.
- Urologist, andrologist in case of ejaculation disorders or Peyronie’s disease.
- Patient group or patient organisation providing peer support.

In cases where there is a suspicion or clinical manifestation of genital GvHD (such as genital changes, urinary symptoms or sexual concerns), patients should be referred either to a gynaecologist, dermatologist, urologist or other clinicians trained in the assessment and management of genital GvHD, for assessment and management of genital GvHD [20].

## Patient information

Assuming that HCPs can provide understandable patient information, many cancer patients cannot recall that sexual changes were discussed, while other patients are not satisfied with the information that was given [15, 16]. The majority of patients prefer verbal and written information [32]. Therefore, this combined approach is preferable.

Earlier work by Gamel *et al* [37] has shown that informational needs regarding sexuality vary across the treatment trajectory. Prior to treatment, the effects of HCT on sexuality should be explained so that, at the time of discharge post HCT, patients may know which sexual activities are restricted and for how long. Awareness of symptoms, monitoring and reporting to HCPs are also important. At the time of rebuilding their sexual life, suggested to be 1 year post HCT [17, 38, 39], patients should be informed about most frequently occurring sexual dysfunction post HCT [37]. During the entire patient information process, patients should be advised that sexual changes are common and given reassurance.

Patients consider medical and treatment information in the context of haematological malignancies of higher priority than psychosocial information [40, 41]. Thus, for most people, sexuality is of minor importance compared to treatment-related issues and survival [40]. However, this only means that the impact of HCT on sexual function is less relevant at that time. As patients return to a ‘normal life’, sexual dysfunction might well become more pressing after the first year post HCT. Patients seem to report impairment of sexual function at earliest 1 year post HCT [17, 38, 39], therefore ideally, from this moment, HCPs should start routinely addressing sexuality.

## Conclusion

The sexual dysfunctions that HCT survivors face are decreased sexual activity, less sexual desire, erectile dysfunction or decreased vaginal lubrication, sexual pain (dyspareunia), orgasm problems and genital changes. Five years post HCT, survivors still report sexual dysfunction. Sexual function of female survivors is not likely to improve without any intervention, whereas sexual function of male survivors might improve within the first 2 years without intervention. HCT survivors need to be informed about the impact that HCT can potentially have on both sexuality and sexual function, because survivors need to be aware of the changes and the support that is available. Patient education can prevent deteriorating sexual function but, without preparatory information, it is more difficult to initiate the required discussions.

## Conflicts of interest

The authors declare that they have no conflicts of interest.

## Funding statement

All of the authors declare that they had no access to funding support for this work.

## Appendix: patient information sheet for patients undergoing HCT: the impact of the disease and treatment on sexual function and sexuality

Sexual dysfunction is a common phenomenon throughout the general population. Often this is influenced by psychological or social factors, but certain chronic diseases can be a direct cause. It is certainly possible that HCT may have an impact on sexual function and related intimacy and sexuality. This may be reflected in negative feelings about one’s body image, self-esteem and sexual relationship.

### What are the acute side effects of the HCT that can affect sexuality?

During the treatment period, the acute side effects of chemotherapy, such as nausea, vomiting, hair loss and fatigue, will most probably lead to a reduction in the desire for sexual activity. Certain treatments, such as chemotherapy with alkylating agents or TBI, can suppress the function of the ovaries and testicles, causing a reduction in male or female hormone production. In men, the low testosterone level can cause problems with erectile function, while in women, premature menopause with vaginal dryness and pain during intercourse. TBI could impair peripheral nerves or pelvic blood flow, causing erectile dysfunction in men or vaginal dryness in women.

### What are the long-term effects of the HCT that can affect sexuality?

Frequently reported problems with sexual function are reduction in sexual desire (often through a negative body-image or not feeling sexually attractive), arousal difficulties (erectile dysfunction in men or vaginal dryness in women), pain during intercourse and absence of orgasm.

Chronic GvHD can lead to various sexual problems in men and women. The first presentation of genital GvHD is often painful sexual intercourse, urinary difficulties and genital skin changes (such as inflammatory and non-inflammatory lesions in men, vulvovaginal dryness/itching/pain/bleeding in women).

### Should sex be avoided?

In order to maintain or re-build a satisfactory sexual relationship between you and your partner, it is important that intimacy and sexual activity is not avoided. There is no medical reason to avoid sexual intercourse as long as this does not cause bleeding or pain. It is realistic that sexuality will change, try not to force but be gentle and use lubricants, especially when platelets are low. Pregnancies do occur after HCT; however, pregnancy should be avoided soon after the treatment. Therefore, it is advised to use contraceptive methods at least until you know what your fertility status is.

### Should sexual problems be reported?

Yes, it is important to report sexual problems to your HCP because in some cases they may indicate medical problems which need to be referred to another medical specialist for treatment. In particular, genital changes or urinary symptoms may indicate GvHD.

### How can sexual problems be managed?

It is important to pay attention to any difficulties as they strongly influence the recovery of the patient. There are several treatment options and several disciplines that can help to manage sexual problems. Some of the examples of treatments are as follows.

- Testosterone and phosphodiesterase type 5 inhibitors [Viagra, Cialis] can improve sexual function in the case of hypogonadism and erectile dysfunction.
- Hormone replacement therapy (HRT) can prevent postmenopausal vaginal atrophy, besides it can also help women experiencing lower desire, orgasmic difficulties and sexual pain. Topical oestrogen can also help vaginal health in addition to non-hormonal moisturisers and lubricants.
- Vaginal lubricants and moisturisers can minimise dryness and pain.

The disciplines that are on hand to help from coping with the problems to actually manage these problems are: nurse practitioners, clinical nurse specialists, social workers, psychologists, psychosexual therapists, pelvic physiotherapists, gynaecologists, dermatologists, urologists, andrologists, sexologists, patient groups or patient organisations.

Sexual problems that occur after HCT are often caused by the disease or the treatment. Early recognition and management of sexual problems can lead to improved sexual function and quality of life for you and your partner. Tell us about it.
